# Supplementary material for: Unbalanced fertilizer use in the Eastern Gangetic Plain: The influence of Government recommendations, fertilizer type, farm size and cropping patterns
Source: PLoS One. 2022 Jul 28;17(7):e0272146. doi: 10.1371/journal.pone.0272146 (PMC9333275; doi:10.1371/journal.pone.0272146)
Supplement: S1 Table — (DOCX) [file pone.0272146.s001.docx]

**S1 Table. Nutrient composition of different nutrient sources**

| **Nutrient Source** | **Nutrient composition (%)** | | | | | | |
| --- | --- | --- | --- | --- | --- | --- | --- |
|  | N | P | K | S | Zn | B | Mg |
| Urea | 46.0 |  |  |  |  |  |  |
| Triple Super Phosphate (TSP) |  | 20.0 |  | 1.30 |  |  |  |
| Diammonium Phosphate (DAP) | 18.0 | 20.0 |  |  |  |  |  |
| Muriate of Potash (MoP) |  |  | 50.0 |  |  |  |  |
| Gypsum |  |  |  | 18.0 |  |  |  |
| MgSO4 |  |  |  | 12.5 |  |  | 9.50 |
| ZnSO4 (mono-hydrate) |  |  |  | 17.5 | 36.0 |  |  |
| ZnSO4 (hepta-hydrate) |  |  |  | 10.5 | 21.0 |  |  |
| Boric acid |  |  |  |  |  | 17.0 |  |
| Cow-dung (decomposed) | 1.0 | 0.30 | 0.46 | 0.36 | 0.15 | 0.011 | 0.44 |
| Poultry manure (decomposed) | 1.25 | 0.70 | 0.95 | 0.48 | 0.013 | 0.013 | 0.80 |
| Vermicompost | 1.61 | 1.02 | 0.73 | 0.89 | 0.16 | 0.015 | 2.60 |
| Rice straw | 0.40 | 0.10 | 1.50 | 0.195 | 0.008 |  | 0.68 |
| Potato haulm | 2.91 | 0.16 | 2.51 |  |  |  |  |
| Maize straw | 1.23 | 0.076 | 0.77 | 0.069 | 0.006 |  | 0.89 |

Source: [15]; [55] and [56]

**Reference**

55. BARI. Annual report (2018-2019). Soil Science Division, Bangladesh Agricultural Research Institute (BARI), Joydebpur, Gazipur-1701. 2019.

56. Singh SO, Kushwah VS, Sharma RC. Effect of fertilizers and residue incorporation on soil fertility in potato based cropping system. J Indian Potato Assoc. 2003; 30: 103–104.
