# Supplementary material for: Unbalanced fertilizer use in the Eastern Gangetic Plain: The influence of Government recommendations, fertilizer type, farm size and cropping patterns
Source: PLoS One. 2022 Jul 28;17(7):e0272146. doi: 10.1371/journal.pone.0272146 (PMC9333275; doi:10.1371/journal.pone.0272146)
Supplement: S2 Table — (DOCX) [file pone.0272146.s002.docx]

**S2 Table. Rates of nutrient inputs (kg ha^-1^) used in the *irrigated rice-fallow-monsoon rice* cropping pattern**

| **Nutrient Source** | **Irrigated Rice** | | | **Monsoon Rice** | | |
| --- | --- | --- | --- | --- | --- | --- |
|  | Mymensingh | Rajshahi | Thakurgoan | Mymensingh | Rajshahi | Thakurgoan |
| **A. Small-scale farm** | | | | | | |
| Urea | 279.8 | 299.4 | 295.9 | 171.2 | 198.8 | 177.2 |
| TSP | 57.8 | 91.4 | 107.7 | 31.6 | 40.2 | 53.8 |
| DAP | 50.7 | 34.5 | 3.5 | 9.6 | 21.3 | 1.2 |
| MoP | 96.2 | 107.3 | 102.6 | 36.2 | 62.3 | 50.9 |
| Gypsum | 14.0 | 24.4 | 13.1 | 4.2 | 16.1 | 3.6 |
| ZnSO4 (Mono-hydrate) | 0.1 | 0.2 | 0.1 | 0.2 | 0.3 | 0.1 |
| ZnSO4 (Hepta-hydrate) | 0.9 | 0.9 | 0.4 | 0.3 | 0.8 | 0.4 |
| MgSO4 |  | 1.7 | 0.6 |  | 0.4 |  |
| Cow-dung | 2422.3 | 2113.0 | 2946.1 | 142.3 | 125.8 | 154.1 |
| Poultry liter | 141.3 |  | 23.5 |  |  |  |
| Vermicompost |  | 99.4 |  |  |  |  |
| **B. Medium-scale farm** | | | | | | |
| Urea | 312.5 | 336.6 | 336.8 | 196.3 | 214.9 | 209.6 |
| TSP | 97.0 | 164.5 | 176.1 | 54.2 | 62.2 | 72.0 |
| DAP | 44.9 | 44.7 | 6.6 | 8.0 | 22.3 | 2.5 |
| MoP | 115.5 | 148.9 | 147.3 | 55.7 | 79.8 | 70.5 |
| Gypsum | 23.6 | 37.7 | 32.3 | 7.6 | 28.1 | 12.1 |
| ZnSO4 (Mono-hydrate) | 2.3 | 2.9 | 2.7 | 0.4 | 0.9 | 0.6 |
| ZnSO4 (Hepta-hydrate) | 1.5 | 0.8 | 0.9 | 1.0 | 1.7 | 0.7 |
| MgSO4 |  | 3.0 | 3.5 |  | 1.3 |  |
| Cow-dung | 1618.8 | 1191.0 | 1776.2 | 63.9 | 79.8 | 51.4 |
| Poultry liter | 59.8 | 162.2 |  | - | - | - |
| Vermicompost | - | 102.8 | - | - | - | - |
| **C. Large-scale farm** | | | | | | |
| Urea | 319.0 | 342.7 | 331.6 | 191.8 | 226.7 | 212.5 |
| TSP | 56.7 | 160.2 | 146.8 | 28.5 | 37.1 | 68.9 |
| DAP | 110.7 | 57.7 | 38.1 | 46.8 | 49.7 | 9.9 |
| MoP | 144.9 | 164.2 | 155.9 | 59.8 | 79.8 | 73.7 |
| Gypsum | 37.1 | 58.0 | 49.9 | 23.7 | 31.2 | 16.7 |
| ZnSO4 (Mono-hydrate) | 2.9 | 3.0 | 2.0 | 0.9 | 1.6 | 1.0 |
| ZnSO4 (Hepta-hydrate) | 3.2 | 2.1 | 3.1 | 2.1 | 1.5 | 1.5 |
| MgSO4 |  | 3.0 | 2.6 |  | 2.3 |  |
| Cow-dung | 836.1 | 1029.2 | 1459.4 |  |  |  |
| Poultry liter | 105.9 |  |  |  |  |  |
| Vermicompost |  | 149.7 |  |  |  |  |
| Irrigated Rice Straw  (aboveground) | 1320.5 | 1038.5 | 1082.4 |  |  |  |
| Monsoon rice straw  (aboveground) |  |  |  | 640.6 | 590.3 | 741.2 |

Source: Field Survey, FGD & KII, 2018-2019.
