# Supplementary material for: Unbalanced fertilizer use in the Eastern Gangetic Plain: The influence of Government recommendations, fertilizer type, farm size and cropping patterns
Source: PLoS One. 2022 Jul 28;17(7):e0272146. doi: 10.1371/journal.pone.0272146 (PMC9333275; doi:10.1371/journal.pone.0272146)
Supplement: S3 Table — (DOCX) [file pone.0272146.s003.docx]

**S3 Table. Rates of nutrient inputs (kg ha^-1^) used in the *potato-maize-monsoon rice* cropping pattern**

| **Nutrient Source** | **Potato** | | **Maize** | | **T. Aman** | |
| --- | --- | --- | --- | --- | --- | --- |
|  | Rajshahi | Thakurgoan | Rajshahi | Thakurgoan | Rajshahi | Thakurgoan |
| **A. Small-scale farm** | | | | | | |
| Urea | 429.1 | 419.8 | 185.2 | 209.2 | 209.3 | 204.8 |
| TSP | 194.4 | 226.1 | 27.6 | 44.5 | 66.8 | 82.9 |
| DAP | 195.8 | 50.5 | 3.7 | 0.0 | 38.9 | 16.8 |
| MoP | 328.6 | 295.3 | 17.4 | 30.9 | 69.4 | 60.3 |
| Gypsum | 84.6 | 62.2 | 7.1 | 19.9 | 9.5 | 4.9 |
| ZnSO4 (Mono-hydrate) | 1.4 | 0.7 | 0.2 | 0.1 | 0.4 | 0.2 |
| ZnSO4 (Hepta-hydrate) | 1.9 | 1.4 | 0.1 | 0.4 | 1.4 | 0.3 |
| Boric Acid | 1.8 | 1.1 | 0.2 | 0.3 |  |  |
| MgSO4 | 12.1 | 8.9 | 1.1 | 0.9 |  |  |
| Cow-dung | 1936.6 | 2896.6 | 88.2 | 210.1 | 46.3 | 42.7 |
| Vermicompost | 14.7 |  |  |  |  |  |
| **B. Medium-scale farm** | | | | | | |
| Urea | 500.9 | 436.3 | 207.0 | 219.8 | 219.7 | 211.0 |
| TSP | 229.0 | 242.8 | 24.8 | 57.6 | 81.0 | 77.8 |
| DAP | 216.7 | 51.9 | 9.9 | 0.0 | 46.2 | 27.9 |
| MoP | 382.8 | 327.3 | 27.3 | 38.2 | 88.8 | 91.6 |
| Gypsum | 96.1 | 77.5 | 19.1 | 35.8 | 16.0 | 8.6 |
| ZnSO4 (Mono-hydrate) | 1.6 | 1.9 | 0.5 | 0.7 | 1.1 | 0.7 |
| ZnSO4 (Hepta-hydrate) | 5.3 | 1.6 | 0.7 | 1.2 | 0.8 | 0.5 |
| Boric Acid | 3.1 | 1.8 | 0.4 | 1.4 |  |  |
| MgSO4 | 25.2 | 14.5 | 1.9 | 3.7 |  |  |
| Cow-dung | 1130.8 | 2165.2 |  | 115.3 |  |  |
| Vermicompost | 18.7 | 0.0 |  |  |  |  |
| **C. Large-scale farm** | | | | | | |
| Urea | 454.5 | 411.3 | 215.2 | 226.8 | 224.6 | 216.1 |
| TSP | 142.6 | 169.8 | 23.4 | 70.8 | 57.6 | 39.5 |
| DAP | 312.9 | 154.4 | 14.0 | 0.0 | 74.5 | 67.3 |
| MoP | 358.3 | 336.5 | 25.8 | 41.2 | 98.2 | 88.7 |
| Gypsum | 105.2 | 77.2 | 19.6 | 28.8 | 23.0 | 12.4 |
| ZnSO4 (Mono-hydrate) | 3.1 | 2.5 | 0.5 | 0.8 | 0.9 | 0.6 |
| ZnSO4 (Hepta-hydrate) | 3.7 | 4.3 | 0.9 | 1.2 | 1.2 | 1.2 |
| Boric Acid | 3.7 | 2.5 | 1.2 | 1.6 |  |  |
| MgSO4 | 28.6 | 21.6 | 4.7 | 3.7 |  |  |
| Cow-dung | 467.8 | 1160.9 |  |  |  |  |
| Vermicompost | 180.0 |  |  |  |  |  |
| Residue Retention  (aboveground) | 180.6 | 220.4 | 340.8 | 390.6 | 444.6 | 518.7 |

Source: Field Survey, FGD & KII, 2018-2019.
