# Supplementary material for: Unbalanced fertilizer use in the Eastern Gangetic Plain: The influence of Government recommendations, fertilizer type, farm size and cropping patterns
Source: PLoS One. 2022 Jul 28;17(7):e0272146. doi: 10.1371/journal.pone.0272146 (PMC9333275; doi:10.1371/journal.pone.0272146)
Supplement: S4 Table — (DOCX) [file pone.0272146.s004.docx]

**S4 Table. Farmers current NPKS use gap in cropping season under different rice-based cropping patterns in the northern Bangladesh**

| **Nutrient** | **Cropping pattern** | | | | | | | | | | | | | |
| --- | --- | --- | --- | --- | --- | --- | --- | --- | --- | --- | --- | --- | --- | --- |
|  | **Irrigated rice-fallow-monsoon rice** | | | | | Nutrient use gap (%) | | **Potato-maize-monsoon rice** | | | | | Nutrient use gap (%) | |
|  | Total Nutrient use  (kg ha^-1^) | Total Recommendation  (kg ha^-1^) | | Nutrient use gap  (kg ha^-1^) | |  |  | Total Nutrient use  (kg ha^-1^) | Total Recommendation  (kg ha^-1^) | | Nutrient use gap  (kg ha^-1^) | |  |  |
|  |  | FRG-2012 | FRG-2018 | FRG-2012 | FRG-2018 | FRG-2012 | FRG-2018 |  | FRG-2012 | FRG-2018 | FRG-2012 | FRG-2018 | FRG-2012 | FRG-2018 |
| **A. Large-scale farm** | | | | | | | | | | | | | | |
| N | 277.6 | 240 | 270 | 37.6 | 7.6 | 15.7 | 2.8 | 464.4 | 360 | 360 | 104.4 | 104.4 | 29.0 | 29.0 |
| P | 57.0 | 30 | 29.7 | 27.0 | 27.3 | 90.0 | 91.9 | 113.2 | 73 | 54 | 40.2 | 59.2 | 55.1 | 109.6 |
| K | 129.5 | 100 | 126 | 29.5 | 3.5 | 29.5 | 2.8 | 244.7 | 165 | 154 | 79.7 | 90.7 | 48.3 | 58.9 |
| S | 20.6 | 30 | 18.7 | -9.4 | 1.9 | -31.3 | 10.2 | 32.9 | 47 | 31.5 | -14.1 | 1.4 | -30.0 | 4.4 |
| **B. Medium-scale farm** | | | | | | | | | | | | | | |
| N | 266.4 | 240 | 270 | 26.4 | -3.6 | 11.0 | -1.3 | 450.9 | 360 | 360 | 90.9 | 90.9 | 25.3 | 25.3 |
| P | 54.1 | 30 | 29.7 | 24.1 | 24.4 | 80.3 | 82.2 | 107.2 | 73 | 54 | 34.2 | 53.2 | 46.8 | 98.5 |
| K | 120.6 | 100 | 126 | 20.6 | -5.4 | 20.6 | -4.3 | 245.8 | 165 | 154 | 80.8 | 91.8 | 49.0 | 59.6 |
| S | 17.2 | 30 | 18.7 | -12.8 | -1.4 | -42.7 | -7.5 | 32.0 | 47 | 31.5 | -15.0 | 0.5 | -31.9 | 1.6 |
| **C. Small-scale farm** | | | | | | | | | | | | | | |
| N | 242.7 | 240 | 270 | 2.7 | -27.3 | 1.1 | -10.1 | 415.8 | 360 | 360 | 55.8 | 55.8 | 15.5 | 15.5 |
| P | 38.8 | 30 | 29.7 | 8.8 | 9.1 | 29.3 | 30.6 | 95.6 | 73 | 54 | 22.6 | 41.6 | 31.0 | 77.0 |
| K | 95.9 | 100 | 126 | -4.1 | -30.1 | -4.1 | -23.9 | 208.9 | 165 | 154 | 43.9 | 54.9 | 26.6 | 35.6 |
| S | 13.2 | 30 | 18.7 | -16.8 | -5.4 | -56.0 | -28.9 | 24.0 | 47 | 31.5 | -23.0 | -7.5 | -48.9 | -23.8 |
| **D. All category** | | | | | | | | | | | | | | |
| N | 252.0 | 240 | 270 | 12.0 | -18.0 | 5.0 | -6.7 | 429.4 | 360 | 360 | 69.4 | 69.4 | 19.3 | 19.3 |
| P | 44.5 | 30 | 29.7 | 14.5 | 14.8 | 48.3 | 49.8 | 100.2 | 73 | 54 | 27.2 | 46.2 | 37.3 | 85.6 |
| K | 105.4 | 100 | 126 | 5.4 | -20.6 | 5.4 | -16.3 | 222.2 | 165 | 154 | 57.2 | 68.2 | 34.7 | 44.3 |
| S | 14.9 | 30 | 18.7 | -15.1 | -3.8 | -50.3 | -20.3 | 27.0 | 47 | 31.5 | -20.0 | -4.5 | -42.6 | -14.3 |
