# Supplementary material for: Unbalanced fertilizer use in the Eastern Gangetic Plain: The influence of Government recommendations, fertilizer type, farm size and cropping patterns
Source: PLoS One. 2022 Jul 28;17(7):e0272146. doi: 10.1371/journal.pone.0272146 (PMC9333275; doi:10.1371/journal.pone.0272146)
Supplement: S5 Table — (DOCX) [file pone.0272146.s005.docx]

**S5 Table. Current nutrient use rates (kg ha^-1^) of different categories of farmers under *irrigated rice-fallow-monsoon rice* cropping pattern.**

| **Farmer Category** | **Irrigated rice** | | | | **Monsoon rice** | | | |
| --- | --- | --- | --- | --- | --- | --- | --- | --- |
|  | Mymensingh | Rajshahi | Thakurgoan | CV (%) | Mymensingh | Rajshahi | Thakurgoan | CV (%) |
| **A. Small-scale farms (n=126)** | | | | | | | | |
| N | 152.1 | 156.5 | 153.1 | 1.5 | 83.8 | 98.0 | 84.7 | 9.0 |
| P | 26.1 | 29.2 | 27.1 | 5.8 | 9.1 | 13.0 | 11.8 | 17.7 |
| K | 59.1 | 63.3 | 63.7 | 4.1 | 28.4 | 39.2 | 36.8 | 16.3 |
| S | 8.7 | 10.7 | 9.9 | 10.3 | 2.8 | 4.8 | 2.7 | 34.5 |
| Zn | 2.1 | 1.9 | 2.3 | 9.5 | 0.3 | 0.4 | 0.3 | 17.3 |
| **B. Medium-scale farms (n=60)** | | | | | | | | |
| N | 161.6 | 171.9 | 166.5 | 3.1 | 94.7 | 105.3 | 99.3 | 5.3 |
| P | 31.3 | 45.0 | 39.6 | 17.9 | 13.2 | 17.5 | 15.5 | 14.0 |
| K | 66.6 | 82.8 | 83.3 | 12.2 | 37.9 | 47.9 | 43.5 | 11.6 |
| S | 9.8 | 13.4 | 13.0 | 16.4 | 3.7 | 7.4 | 4.4 | 38.0 |
| Zn | 2.4 | 2.2 | 2.5 | 6.5 | 0.5 | 0.8 | 0.4 | 36.7 |
| **C. Large-scale farms (n=12)** | | | | | | | | |
| N | 172.8 | 175.6 | 168.2 | 2.2 | 99.3 | 115.3 | 101.7 | 8.2 |
| P | 35.4 | 46.2 | 39.5 | 13.5 | 15.7 | 17.9 | 16.3 | 6.8 |
| K | 79.7 | 89.5 | 86.9 | 5.9 | 39.8 | 47.7 | 45.0 | 9.1 |
| S | 10.6 | 16.7 | 15.2 | 22.4 | 6.3 | 7.6 | 5.3 | 18.0 |
| Zn | 2.4 | 2.4 | 2.5 | 2.4 | 0.8 | 0.9 | 0.7 | 12.5 |

Note: CV denotes coefficient of variation.
