# Supplementary material for: Unbalanced fertilizer use in the Eastern Gangetic Plain: The influence of Government recommendations, fertilizer type, farm size and cropping patterns
Source: PLoS One. 2022 Jul 28;17(7):e0272146. doi: 10.1371/journal.pone.0272146 (PMC9333275; doi:10.1371/journal.pone.0272146)
Supplement: S6 Table — (DOCX) [file pone.0272146.s006.docx]

**S6 Table. Current nutrient use rates (kg ha^-1^) of different categories of farmers under *potato-maize-monsoon rice* cropping pattern in the selected study areas.**

| **Farmer Category** | **Potato** | | | **Maize** | | | **Monsoon rice** | | |
| --- | --- | --- | --- | --- | --- | --- | --- | --- | --- |
|  | Rajshahi | Thakurgoan | CV  (%) | Rajshahi | Thakurgoan | CV  (%) | Rajshahi | Thakurgoan | CV  (%) |
| **A. Small-scale farms** | | | | | | | | | |
| N | 233.5 | 203.2 | 9.8 | 88.9 | 100.5 | 8.7 | 105.6 | 99.9 | 3.9 |
| P | 78.3 | 55.6 | 24.0 | 6.5 | 9.4 | 25.8 | 21.4 | 20.2 | 4.1 |
| K | 167.6 | 151.5 | 7.1 | 11.2 | 18.7 | 35.5 | 36.1 | 31.7 | 9.2 |
| S | 20.1 | 16.0 | 16.1 | 2.0 | 4.7 | 57.0 | 3.0 | 2.2 | 21.8 |
| Mg | 2.7 | 2.6 | 2.7 | 0.1 | 0.1 | 0.0 |  |  |  |
| Zn | 0.9 | 0.6 | 28.3 | 0.2 | 0.3 | 28.3 | 0.5 | 0.2 | 60.6 |
| B | 0.3 | 0.2 | 28.3 |  |  |  |  |  |  |
| OM  (t ha^-1^) | 2.0 | 2.9 | 26.0 |  |  |  |  |  |  |
| **B. Medium-scale farms** | | | | | | | | | |
| N | 270.3 | 211.1 | 17.4 | 99.6 | 104.9 | 3.7 | 111.5 | 104.5 | 4.6 |
| P | 89.4 | 59.2 | 28.7 | 7.1 | 11.9 | 35.7 | 25.6 | 21.3 | 13.0 |
| K | 194.7 | 167.5 | 10.6 | 15.9 | 22.1 | 23.1 | 45.7 | 47.3 | 2.4 |
| S | 24.7 | 19.9 | 15.2 | 4.2 | 8.1 | 44.8 | 4.3 | 2.9 | 27.5 |
| Mg | 3.9 | 3.1 | 16.2 | 0.2 | 0.4 | 47.1 |  |  |  |
| Zn | 1.7 | 1.1 | 30.3 | 0.3 | 0.6 | 47.1 | 0.6 | 0.4 | 28.3 |
| B | 0.5 | 0.3 | 35.4 |  |  |  |  |  |  |
| OM  (t ha^-1^) | 1.1 | 2.2 | 47.1 |  |  |  |  |  |  |
| **C. Large-scale farms** | | | | | | | | | |
| N | 266.3 | 218.0 | 14.1 | 104.2 | 107.5 | 2.2 | 118.8 | 113.9 | 3.0 |
| P | 91.3 | 65.1 | 23.7 | 7.6 | 14.3 | 43.3 | 26.5 | 21.5 | 14.7 |
| K | 182.5 | 172.2 | 4.1 | 15.2 | 23.3 | 29.8 | 50.4 | 45.9 | 6.6 |
| S | 25.6 | 20.2 | 16.7 | 4.6 | 6.8 | 27.3 | 5.3 | 3.1 | 37.0 |
| Mg | 4.2 | 3.8 | 7.1 | 0.4 | 0.4 | 0.0 |  |  |  |
| Zn | 1.9 | 1.8 | 3.8 | 0.4 | 0.6 | 28.3 | 0.6 | 0.5 | 12.9 |
| B | 0.6 | 0.4 | 28.3 |  |  |  |  |  |  |
| OM  (t ha^-1^) | 0.6 | 1.2 | 47.1 |  |  |  |  |  |  |

Note: CV denotes coefficient of variation.
