# Supplementary material for: Unbalanced fertilizer use in the Eastern Gangetic Plain: The influence of Government recommendations, fertilizer type, farm size and cropping patterns
Source: PLoS One. 2022 Jul 28;17(7):e0272146. doi: 10.1371/journal.pone.0272146 (PMC9333275; doi:10.1371/journal.pone.0272146)
Supplement: S7 Table — (DOCX) [file pone.0272146.s007.docx]

**S7 Table. Recommended nutrient rates (kg ha^-1^) of irrigated rice, monsoon rice, potato and maize grown under *irrigated rice-fallow-monsoon rice* cropping pattern and *potato-maize-monsoon rice* cropping patterns**

| **Nutrient** | **Irrigated rice-fallow-monsoon rice** | | | | | |
| --- | --- | --- | --- | --- | --- | --- |
|  | **Irrigated rice (Yield goal: 7.5 ± 0.75 t ha^-1^)** | | | | | |
|  | FRG-2012 | | | FRG-2018 | | |
|  | Mymensingh | Rajshahi | Thakurgoan | Mymensingh | Rajshahi | Thakurgoan |
| N | 150 | 150 | 150 | 180 | 180 | 180 |
| P | 20 | 20 | 20 | 24 | 24 | 16 |
| K | 65 | 65 | 65 | 76 | 76 | 76 |
| S | 18 | 18 | 18 | 12 | 12 | 18 |
| Zn | 1.3 | 1.3 | 1.3 | 1.5 | 2.5 | 1.3 |
|  | **Monsoon rice (Yield goal: 5.0 ± 0.5 t ha^-1^)** | | | | | |
| N | 90 | 90 | 90 | 90 | 90 | 90 |
| P | 10 | 10 | 10 | 10 | 8.0 | 7.0 |
| K | 35 | 35 | 35 | 50 | 50 | 50 |
| S | 12 | 12 | 12 | 4.0 | 4.0 | 6.0 |
| Zn | 1.0 | 1.0 | 1.0 | 1.0 | 1.5 | 1.0 |
|  | **Potato-maize-monsoon rice** | | | | | |
|  | **Potato (Yield goal: 30.0 ± 3.0 t ha^-1^)** | | | | | |
| N |  | 135 | 135 |  | 135 | 135 |
| P |  | 30 | 30 |  | 30 | 20 |
| K |  | 90 | 90 |  | 90 | 90 |
| S |  | 10 | 10 |  | 10 | 15 |
| Zn |  | 2.0 | 2.0 |  | 4.0 | 0.0 |
| B |  | 1.0 | 1.0 |  | 1.0 | 1.5 |
| Mg |  | 5.0 | 5.0 |  | 0.0 | 5.0 |
| OF (t ha^-1^) |  | 5.0 | 5.0 |  | 3.0 | 3.0 |
|  | **Maize (Yield goal: 6.0 ± 0.6 t ha^-1^)** | | | | | |
| N |  | 135 | 135 |  | 135 | 135 |
| P |  | 33 | 33 |  | 23 | 23 |
| K |  | 45 | 45 |  | 31 | 31 |
| S |  | 27 | 27 |  | 14 | 14 |
| Zn |  | 2.0 | 2.0 |  | 2.0 | 2.0 |
| Mg |  | 0.0 | 5.0 |  | 0.0 | 5.0 |
|  | **Monsoon rice (Yield goal: 5.0 ± 0.5 t ha^-1^)** | | | | | |
| N |  | 90 | 90 |  | 90 | 90 |
| P |  | 10 | 10 |  | 6 | 6 |
| K |  | 30 | 30 |  | 33 | 33 |
| S |  | 10 | 10 |  | 6.0 | 6.0 |
| Zn |  | 1.0 | 1.0 |  | 1.0 | 1.0 |
