# Supplementary material for: Unbalanced fertilizer use in the Eastern Gangetic Plain: The influence of Government recommendations, fertilizer type, farm size and cropping patterns
Source: PLoS One. 2022 Jul 28;17(7):e0272146. doi: 10.1371/journal.pone.0272146 (PMC9333275; doi:10.1371/journal.pone.0272146)
Supplement: Attachment — Submitted filename: Response to Reviewers.docx [file pone.0272146.s009.docx]

**Response to Reviewers` Comments**

**30 April 2022**

**Journal Name: PLOS ONE**

**Manuscript Number:** PONE-D-22-01407

**Manuscript Title:** Unbalanced Fertilizer Use in the Eastern Gangetic Plain: the Influence of Government Recommendations, Fertilizer Type, Farm Size and Cropping Patterns

| **No** | **Comments from Academic Editor and the Reviewers** | **Response to Reviewer** |
| --- | --- | --- |
|  | **Academic Editor** |  |
|  | This research paper describes the reason behind the yield gap in Bangladesh. Please address the following issues:   (i) to improve the synchronization between the paragraphs in Introduction, (ii) ambiguity of cropping system is to be addressed, (iii) (Results need to be more specific and irrelevant information may be omitted in Results and Discussion, (iv) The authors should highlight the reason behind the higher P application (overdose) by small and marginal farmers. Also, the reason behind 16-90% over dose of NPK. (v) Lack of more detailed information about the approaches by the Government. | (i) We have added some sentences in the introduction section (line 57-58, line 74-79 and line 85-88) which improve the readability and linkages between the paragraphs in this section.  (ii) Cropping year convention used in Bangladesh consists of three major seasons - Robi season (mid-November to mid-March), Kharif-1 season (mid-March to mid-July) and Kharif-2 (mid-July to mid-November) season. The FRG sequence of crops in the cropping pattern always list the Robi season crop as first crop, so we have followed that convention. Please see the following reference which published by PLOS ONE journal: Al Mamun M.A, Nihad SAI, Sarkar M.AR, Aziz M.A, Qayum M.A, Ahmed R, et al. (2021) Growth and trend analysis of area, production and yield of rice: A scenario of rice security in Bangladesh. PLoS ONE 16(12): e0261128. doi.org/10.1371/journal.pone.0261128  (iii) We have re-written the results section as per reviewer suggestions and omitted irrelevant information from the manuscript.  (iv) The reason behind the higher P application (overdose) by different farm sizes including small-scale farms and 16-90% over dose of NPK is described thoroughly in the sub-section 4.3.  (v) Lack of more detailed information about the approaches by the Government is presented in the sub-section 4.3, line 476-480. |
|  | **Reviewer-1** |  |
| 1 | The manuscript "Unbalanced Fertilizer Use in the Eastern Gangetic Plain: the Influence of Government Recommendations, Fertilizer Type, Farm Size and Cropping Patterns" is written well. It is interesting and useful for researchers, farmers and policy makers of Bangladesh. I recommend to improve the figures quality. | We have changed all the previous figures of the manuscripts. |
|  | **Reviewer-2** |  |
| 1 | Farmers generally apply fertilizer based on crop requirement, market price, subsidy, climate etc. The fact that FRG 18 has higher values for NPK than 12 indicate that there were requirements for higher NPK doses for crops. Further two FRG guidelines created confusion, you must focus on only one. | The variation in nutrient gaps among the two FRGs was an important factor in this study. We have justified (sub-section 2.5) why we assessed gaps relative to both FRGs. The FRG-2012 recommendation was available for the farmers at the time of the survey. But the FRG-2012 was replaced by a new version of FRG (FRG-2018) which was available for the farmers soon after the field survey was completed. To assess the existing scenario of nutrient use gaps and to identify future nutrient use gaps we assessed both FRGs. |
| 2 | If we consider farmers wise, they will apply fertilizer until they will get some good response, else not. However, they may apply more of one and less of another based on availability, crop response and amount to be invested. If there is subsidy on fertilizer, why farmers applied less of micronutrients and K. I am surprised to see that even small and marginal farmers applied over dose of P fertilizer. Explain what could be the reason? | This is another very important question. We have explained the reason of over-dosing and under-dosing of nutrients (including micronutrients) in the sub-section 4.3. Generally, both farmers’ perception and fertilizer subsidy are key matters in deciding fertilizer rates. Farmers perception “higher P provides higher yield” along with recent increase subsidy on P fertilizers is highly responsible for P overdosing even by the small and marginal farmers. On the other hand, lack of awareness was main barrier to optimal K use especially in rice crops. During focus group discussion (FGD), farmers reported that they are unable to use micronutrients due to higher price. Currently Govt. is providing subsidy only for NPK containing fertilizers. |
| 3 | You told that there were in general 16-90% over dose of NPK used by different categories of farmers? What's their proportionate yield and income enhancement? | In the present study, we defined three categories of farmers: over-user, under-user and recommended dose user and then estimated the crop yield and farm income difference among different levels of nutrient user (Table 5 and Table 6). Due to small sample size especially for large and medium farmers, we did not estimate yield and income enhancement due to recommended nutrient dose use by farm sizes with nutrient use gaps (over-user and under-user). Since this was a survey-based study, the data collected was not suitable to estimate proportionate yield and income enhancement for individual nutrient over-dosing and under-dosing. But, our findings clearly indicated that Govt. endorsed recommended dose user received higher yield and income over unbalanced nutrient user (over-doing or under-dosing). |
| 4 | Whether subsidy on fertilizer is blanket? Or it’s based on certain principles say for a farmers cultivating X ha land will he get Y amount subsidy? Or its subsidy on all amount they want to apply? | In Bangladesh, Govt. follows universal subsidy policy which means price of NPK-containing fertilizers (per kilogram) is same for all categories of farmers what amount they wish to purchase (mentioned in the sub-section 4.1, line 407-412). |
| 5 | You mentioned in line no 474 that there is lack of trust on Govt. recommendation? What could be the reason? How it’s addressed by the concerned Dept.? What’s the novelty of the study and also scientific basis? I find it’s more of a technology Gap or lack of awareness among farmers due to inappropriate extension activities. There should be pilot scale demonstrations on recommended doses and the farmers should be made aware by their visit and hands on in such study areas. | During FGD, farmers reported that they think Government nutrient recommendation is too conservative and they can enhance crop yield by increasing the rates of nutrients. The Governments various extension activities related to modern technologies adoption including recommended fertilizer use often remain ineffective because of less focus on the large numbers of the small and marginal farms that make the majority of farmers. In a related pilot scale project, we are engaging with farmers on recommended fertilizer doses to replace current practices with balanced nutrients by building their confidence. The existing weaknesses in the extension system will be made more apparent by the present results in the study areas. We have discussed these issues in the sub-section 4.3.  The research gaps are now presented in the introduction section and the novelty of study is presented in the discussion section (last paragraph of sub-section 4.1, line 422-427). |
| 6 | A detail and critical analysis on impact of over or less use is required on productivity, income, environmental pollution and Govt. schemes on subsidy for its future rationalization. | A detailed and critical analysis on impact of over or less use is presented in the sub-section 4.5. We have re-arranged the sub-section based on the comments of Reviewer-3. |
| 7 | Whether Govt. recommendation is based on STCR (soil test crop response), or any other scientific approaches? | Govt. recommendation is based on STCR (soil test crop response). |
|  | **Reviewer-3** |  |
| 1 | The introduction provides sufficient background information. However, it lacks synchrony between the paragraphs. Please improve readability and linkages between the paragraphs in this section. | We have added some sentences in the introduction section that improve readability and linkages between the paragraphs in this section. |
| 2 | There is no clear indication of objectives; instead, the authors merely highlight what they were intended to.  Please check the sequence of citations……..it starts with [8]! | The aim of the study (including hypothesis) is now stated in the last paragraph of the introduction section. We have re-arranged the sequence of citations. |
| 3 | M&M I find ambiguity in the represented sequence of crops in the cropping patterns. A cropping pattern should represent the crops cultivated from beginning of a calendar year not from the end of it. For example, monsoon rice is always cultivated ahead of irrigated rice in a calendar year; so, the cropping pattern should represent as: monsoon rice-irrigated rice-fallow. The other cropping pattern should be: maize (summer)-monsoon rice (rainy)-potato (winter). I suggest to comply with it in whole MS. | Cropping year convention used in Bangladesh consists of three major seasons - Robi season (mid-November to mid-March), Kharif-1 season (mid-March to mid-July) and Kharif-2 (mid-July to mid-November) season. The FRG sequence of crops in the cropping pattern always list the Robi season crop as first crop, so we have followed that convention.  Recent articles published by PLOS ONE followed the same convention for sequence of crops under a cropping pattern (e.g., irrigated rice (called as Boro rice)-fallow-monsoon rice (called as T. Aman rice).  Al Mamun M.A, Nihad SAI, Sarkar M.AR, Aziz M.A, Qayum M.A, Ahmed R, et al. (2021) Growth and trend analysis of area, production and yield of rice: A scenario of rice security in Bangladesh. PLoS ONE 16(12): e0261128. doi.org/10.1371/journal.pone.0261128 |
| 4 | L94-102: Please provide grid information for the experimental sites. | We have provided grid information for the study sites in the first para of sub-section 2.1. |
| 5 | L100: Add citation to the sampling procedure. | We have added citation to the sampling procedure. |
| 6 | Descriptive statistics: Sentences are very clumsy with poor readability. Please recast sentences to improve clarity. In a few instances, results are discussed (e.g., L340-342), which should be avoided. | We have re-written the sentences to improve clarity. |
| 7 | L371-393: Unnecessary paragraphs and should be omitted. A part may go to M&M section. Why have the authors placed Table 5 in discussion section? It should go to M&M section. | Most of the unnecessary paragraphs (L371-393) are omitted from the discussion section. We now placed the Table 5 in the result section (Sub-section 3.1) as it is a finding of our present study. |
| 8 | L433-436: The authors must not confuse between HYVs and hybrids! Be precise. | We have rearranged the sentences |
| 9 | L576-591: This part of discussion seems unnecessary. These are fallout of the imbalanced fertilizer uses and a part of it is already highlighted in introduction. I would suggest to erase this part, along with environmental loss scenario in heading. | As per your suggestion, we have discarded the paragraph (L576-591) and re-written the headings. |
