# Supplementary material for: Unbalanced fertilizer use in the Eastern Gangetic Plain: The influence of Government recommendations, fertilizer type, farm size and cropping patterns
Source: PLoS One. 2022 Jul 28;17(7):e0272146. doi: 10.1371/journal.pone.0272146 (PMC9333275; doi:10.1371/journal.pone.0272146)
Supplement: Attachment — Submitted filename: Response to Reviewers.docx [file pone.0272146.s010.docx]

**Response to Reviewers` Comments**

**30 June 2022**

**Journal Name: PLOS ONE**

**Manuscript Number:** PONE-D-22-01407R1

**Manuscript Title:** Unbalanced Fertilizer Use in the Eastern Gangetic Plain: the Influence of Government Recommendations, Fertilizer Type, Farm Size and Cropping Patterns

| **No** | **Comments from Academic Editor and the Reviewers** | **Response to Reviewer** |
| --- | --- | --- |
|  | **Academic Editor** |  |
|  | Please review your reference list to ensure that it is complete and correct. If you have cited papers that have been retracted, please include the rationale for doing so in the manuscript text, or remove these references and replace them with relevant current references. Any changes to the reference list should be mentioned in the rebuttal letter that accompanies your revised manuscript. If you need to cite a retracted article, indicate the article’s retracted status in the References list and also include a citation and full reference for the retraction notice. | We have re-checked the reference list and found all are in the reference list which we have cited in the manuscript. We have included some references (e.g. reference 54) as well as replaced some references (e.g. reference 10) in order to emphasise regional (Eastern Gangetic Plain) context as well as address the reviewers’ comments and suggestions. |
|  | **Reviewer-2** |  |
| 1 | You must mention some assessment about how much is yield or income enhancement due to overuse of fertilizer over the recommended one by the govt.? | Yield and income enhancement of different levels of nutrient user, due to balanced fertilizer use, are shown in Table 5 and Table 6, respectively which were testing for significance by the t-test. Recommended nutrient user received significant higher yield and income over under-user and over-user category. Only potato over-user famers got slightly higher yield over recommended user but it was statistical non-significant. We discussed these topics in the discussion section (line 559-571). |
| 2 | Also give your own assessment the impact of blanket subsidy to all group of farmers and also on possible enhancement in productivity, food security and economical impact. | Our own assessment of the impact of blanket subsidy (both Govt. and farmers perspective) to all group of farmers is mentioned in the discussion section (line 572-586). The blanket fertilizer subsidy has increased crop productivity and farming efficiency but requires substantial investment by the Governments. Increased productivity of rice crop has positive impact on achieving food security. If Govt. removed the blanket fertilizer subsidy, the food price could rise dramatically. That’s why our suggestion was to implement balanced fertilizer use practices by the farmers that could save Govt. treasury as well as ensure food security by increasing crop productivity and farming profitability. |
